# Hartshorne’s Conspectus of Medical Sciences

**Published:** 1874-09

**Authors:** 


A CONSPECTUS OF THE MEDICAL SCIENCES: comprising Manuals of Anatomy, Physiology, Chemistry, Materia Medica, Practice of Medicine, Surgery and Obstetrics. For the use of students. By Henry Hartshorne, A.M., M.D., Professor of Hygiene in the University of Pennsylvania, etc. Second edition, enlarged and thoroughly revised, with 477 illustrations. Philadelphia: Henry C. Lea, 1874. 12 mo., leather, pp. 1024. Price, $5.00; cloth $4.25.

We are not of those who decry medical compendiums for the use of students. When properly compiled and properly used, they fill a valuable place in recalling to the mind and fixing upon the memory the more salient truths of medical science. That they may be, and are, used in the process of cramming for the green-room is not to be denied; and yet the discrimination between those who are thus crammed and the the real student, who has feasted leisurely upon true knowledge, and properly digested and appropriated it, is not difficult to make.

The work before us has already successfully asserted its claim to the confidence and favor of the profession; it but remains for us to say that in the present edition the whole work has been fully overhauled and brought up to the present status of the science. Upon several points of the doctrine taught we might offer some criticisms, but the general excellence of the work leads us to rest satisfied with it as it is. The work of the publisher is well done, and the binding is strong and durable, as it ought to be.

